# Healthcare-associated infections and antimicrobial resistance in Canadian acute care hospitals, 2020–2024

**DOI:** 10.14745/ccdr.v52i05a05

**Published:** 2026-05-31

**Authors:** Canadian Nosocomial Infection Surveillance Program

**Affiliations:** 1 Centre for Communicable Diseases and Infection Control, Public Health Agency of Canada, Ottawa, ON

**Keywords:** healthcare-associated infections, community-associated infections, antimicrobial resistance, surveillance, *Clostridioides difficile* infection, methicillin-resistant *Staphylococcus aureus*, vancomycin-resistant *Enterococcus*, carbapenemase-producing *Enterobacterales*, *Escherichia coli*, *Candidozyma auris*, Canadian Nosocomial Infection Surveillance Program

## Abstract

**Background:**

Healthcare-associated infections (HAIs) and antimicrobial resistance (AMR) continue to contribute to excess morbidity and mortality among Canadians.

**Objective:**

To describe epidemiologic and laboratory characteristics and trends of HAIs and AMR, 2020–2024, using surveillance and laboratory data submitted by hospitals to the Canadian Nosocomial Infection Surveillance Program (CNISP) and by provincial and territorial laboratories to the National Microbiology Laboratory.

**Methods:**

Data was collected from 109 Canadian sentinel acute care hospitals between January 1, 2020 and December 31, 2024 for *Clostridioides difficile* infections (CDI), methicillin-resistant *Staphylococcus aureus* (MRSA) bloodstream infections (BSIs), vancomycin-resistant *Enterococcus* (VRE) BSIs (specifically *Enterococcus faecalis* and *Enterococcus faecium*), carbapenemase-producing *Enterobacterales* (CPE) and carbapenemase-producing *Acinetobacter baumannii* (CPA) infections and colonizations and *Candidozyma auris* (*C. auris*; formerly *Candida auris*) infections. Trend analysis for case counts, incidence rates (rates), outcomes, molecular characterization and AMR profiles are presented.

**Results:**

From 2020 to 2024, rates remained relatively stable for CDI (range: 5.01–5.38 infections per 10,000 patient days) and MRSA BSI (range: 0.99–1.16 infections per 10,000 patient days) and increased significantly for VRE BSIs (from 0.30 to 0.42 infections per 10,000 patient days; *p*=0.01). During this time, infection rates for CPE remained low compared to other HAIs but increased significantly (rates: 0.05–0.20; *p*=0.03), CPA counts continue to remain very low (n=22 infections) and *C. auris* counts remained low compared to other HAIs (n=43 isolates).

**Conclusion:**

The incidence of MRSA BSIs and CDI remained stable and VRE BSIs and CPE infections increased in the Canadian acute care hospitals participating in CNISP. An increased number of *C. auris* isolates were identified. Reporting standardized surveillance data to inform the application of infection prevention and control practices in acute care hospitals is critical to help decrease the burden of HAIs and AMR in Canada.

## Introduction

Though often preventable, healthcare-associated infections (HAIs) represent one of the most common adverse events experienced by patients in acute care settings globally ((1)). In addition to increasing morbidity and mortality, HAIs are associated with longer lengths of stay in hospitals and higher costs of care ((1)). In Canada, a point prevalence survey conducted in 2024 estimated that the prevalence of patients with at least one HAI was 8.1% ((2)). The prevalence of HAIs in 2019–2023 has been estimated to be 7.6% in England, 8.0% in Europe and 9.9% in Australia ((3–5)).

Many microorganisms responsible for HAIs exhibit high levels of antimicrobial resistance (AMR), and rising resistance rates threaten progress in reducing HAI incidence ((6)). Infections caused by resistant organisms carry an estimated 85% higher risk of death compared to infections by susceptible organisms, and in 2019, bacterial AMR infections were linked to roughly five million deaths worldwide ((7,8)). Evidence from Canada and other countries demonstrates that healthcare-associated (HA) methicillin-resistant *Staphylococcus aureus* (MRSA) bloodstream infections (BSIs) lead to substantial morbidity and mortality, longer hospitalizations and increased healthcare costs ((9–12)). The prevalence of AMR is projected to reach 40% by 2050 ((13)). Under this scenario, an estimated 13,700 Canadians could die annually from resistant infections, with an associated economic burden of $21 billion per year to Canada’s Gross Domestic Product (GDP) ((13)). In addition, newly emerging resistant organisms, such as *Candidozyma auris* (*C. auris*; formerly *Candida auris*), have prompted the need for strengthened surveillance and revisions to existing infection prevention and control practices ((14)).

In Canada, the Public Health Agency of Canada (PHAC) collects national data on various HAIs and AMR through the Canadian Nosocomial Infection Surveillance Program (CNISP). In line with the World Health Organization (WHO)’s core components of infection prevention and control ((15)), CNISP performs consistent, standardized surveillance to reliably estimate HAI burden, establish benchmark rates for national and international comparison, identify potential risk factors and assess and inform specific interventions to improve patient health outcomes. Data provided by CNISP directly support the collaborative goals outlined in the *Pan-Canadian Action Plan on Antimicrobial Resistance* ((16)) and provides vital information on many of the AMR pathogens included in Canada’s 2025 priority antimicrobial-resistant pathogens list ((17)).

This report describes the most recent HAI and AMR surveillance data collected from CNISP participating hospitals between 2020 and 2024.

## Methods

### Design

Established in 1994, CNISP is a collaboration between the PHAC, the Association of Medical Microbiology and Infectious Disease Canada and sentinel hospitals from across Canada. The goal of CNISP is to facilitate and inform the prevention, control and reduction of HAIs and AMR organisms in Canadian acute care hospitals through active surveillance and reporting. The CNISP conducts prospective, sentinel surveillance for HAIs (including AMR organisms) ((18)).

### Case definitions

Standardized case definitions for HA and community-associated (CA) infections were used. The 2024 surveillance case definition and eligibility criteria are available upon request from the author.

### Data sources

Between January 1, 2020 and December 31, 2024, participating hospitals submitted epidemiologic data and isolates for cases meeting the respective case definitions for *Clostridioides difficile* infections (CDIs), MRSA BSIs, vancomycin-resistant *Enterococcus* (VRE) BSIs (specifically *Enterococcus faecalis* and *Enterococcus faecium*), carbapenemase-producing *Enterobacterales* (CPE) and carbapenemase-producing *Acinetobacter baumannii* (CPA) (infections or colonizations). *Candidozyma auris* isolates (infections or colonizations) were identified by provincial and territorial laboratories and participating hospital laboratories.

In 2024, 109 hospitals in 10 provinces and one territory participated in HAI surveillance and are further described in **Table 1**. Hospital participation varied by surveillance project and year (Supplemental material is available upon request from the author). In 2024, CNISP HAI surveillance, patient admissions were categorized according to hospital bed size; small (1–200 beds, n=56 sites, 51.3%), medium (201–499 beds, n=33 sites, 30.3%) or large (500 or more beds, n=20 sites, 18.3%). Overall, 44 sites (40%) were in Western Canada (British Columbia, Alberta, Saskatchewan and Manitoba), 38 (35%) were in Central Canada (Ontario and Québec), 26 (24%) were in Eastern Canada (Nova Scotia, New Brunswick, Prince Edward Island and Newfoundland and Labrador) and one (0.9%) was in Northern Canada (Yukon, Northwest Territories and Nunavut) (Table 1). In addition to adult, mixed and paediatric hospital types reported in previous years, CNISP added two additional categories of hospital type in 2024. Adult-neonatal intensive care unit (Adult-NICU) and paediatric-obstetric (Paediatric-OB) were added as hospital types to better characterise the patient population served by those facilities.

**Table 1:** Summary of hospitals participating in the Canadian Nosocomial Infection Surveillance Program, by region, 2024

Hospital characteristics	Region				Total
	Western^a^	Central^b^	Eastern^c^	Northern^d^	
Total number of hospitals	44	38	26	1	109
**Hospital type**					
Adult^e^	20	15	16	0	51
Adult-NICU	2	6	0	0	8
Mixed^f^	17	11	8	1	37
Paediatric^g^	4	4	1	0	9
Paediatric-OB	1	2	1	0	4
**Hospital size**					
Small (1–200 beds)	20	13	22	1	56
Medium (201–499 beds)	15	15	3	0	33
Large (500 or more beds)	9	10	1	0	20
**Admissions and discharge**					
Total number of beds	13,164	13,429	3,207	26	29826
Total number of admissions	634,776	583,028	112,601	1,935	1,332,340
Total number of patient days	5,148,759	4,455,471	1,038,466	6,405	10,649,101

Abbreviations: NICU, neonatal intensive care unit; OB, obstetric

^a^ Western refers to British Columbia, Alberta, Saskatchewan and Manitoba

^b^ Central refers to Ontario and Québec

^c^ Eastern refers to Nova Scotia, New Brunswick, Prince Edward Island and Newfoundland and Labrador

^d^ Northern refers to Yukon, Northwest Territories and Nunavut

^e^ Adult standalone hospitals excluding adult facilities with a neonatal intensive care unit

^f^ Mixed hospitals provide both adult and paediatric care

^g^ Paediatric standalone hospitals excluding mixed facilities with women’s and obstetric wards

Epidemiologic (demographic, clinical and outcomes) and denominator data (patient days and patient admissions) were collected and submitted by participating hospitals through the Canadian Network for Public Health Intelligence—a secure online data platform.

Reviews of standardized protocols and case definitions are conducted annually by established infectious disease expert working groups; training for data submission is provided to participating CNISP hospital staff as required. Data quality for surveillance projects is periodically evaluated; additional details on the methodology have been published previously ((19,20)).

### Laboratory data

All patient-linked laboratory isolates (stool samples for CDI cases) were sent to the PHAC’s National Microbiology Laboratory for molecular characterization and antimicrobial susceptibility testing. Isolates for MRSA BSIs, VRE BSIs, CPE/CPA (infections or colonizations), *C. auris* (infections/colonizations) and paediatric CDIs were submitted year-round. Adult CDI isolates were submitted annually during a targeted two-month period (March 1 to April 30).

### Statistical analysis

Rates of HAI were calculated by dividing the total number of cases identified in patients admitted to CNISP participating hospitals by the total number of patient admissions (multiplied by 1,000) or patient days (multiplied by 10,000). Due to low case numbers, rates for *C. auris* and CPA were not calculated. The HAI rates are reported nationally and by region. Due to the low number of CA-VRE BSI cases reported each year, stratified rates as well as mortality rates and laboratory results for CA-VRE BSIs were not included in this report. Sites that were unable to provide case data were excluded from rate calculations and missing denominator data were estimated using their previous years reported data, where applicable. Missing epidemiological and molecular data were excluded from analysis. The Mann-Kendall test was used to assess monotonic trends in rates over time. The chi-square test for trend was used to analyze trends in proportions over time. The chi-square test was used to compare two categorical variables, while the t-test was used to compare differences between groups. Significance testing was two-tailed and differences were considered significant at *p*≤0.05. The stability of rates over time indicates that there was no statistically significant trend observed. Where available, all-cause mortality were reported for HAIs. All-cause mortality rate was defined as the number of deaths per 100 HAI cases 30 days following positive culture.

## Results

### *Clostridioides difficile* infection

Between 2020 and 2024, overall CDI rates remained stable, ranging from 5.01 to 5.38 infections per 10,000 patient days. The rates appeared to decline from 5.38 in 2020 to 5.01 in 2022, followed by a modest increase to 5.19 in 2024; however, no significant trend was observed (*p*=0.81) (**Table 2**). The median age among CDI patients was 69 years (interquartile range [IQR]: 56–79), with males and females each representing 50.3% of the total cases (Supplemental material).

**Table 2:** *Clostridioides difficile* infection data, Canada, 2020–2024^a^

*C. difficile* infection data	Number of infections and incidence rates (per year)				
	2020	2021	2022	2023	2024
**All cases**					
Number of *C. difficile* infection cases	3,649	3,639	3,877	4,736	4,818
Rate per 1,000 patient admissions	4.16	3.97	4.18	4.16	4.17
Rate per 10,000 patient days	5.38	5.07	5.01	5.21	5.19
Number of reporting hospitals	81	81	81	99	97
**All-cause mortality rate**					
Number of deaths	43	66	64	76	76
All-cause mortality rate per 100 cases (%)^b^	9.0	8.8	8.9	8.2	8.0
**HA-CDI**					
Number of HA-CDI cases	2,624	2,570	2,818	3,310	3,409
Rate per 1,000 patient admissions	3.05	2.85	3.09	2.96	2.99
Rate per 10,000 patient days	3.89	3.60	3.66	3.66	3.69
Number of reporting hospitals	81	81	81	99	97
**All-cause mortality rate**					
Number of deaths	39	50	54	59	58
All-cause mortality rate per 100 cases (%)^b^	8.7	9.2	9.9	8.6	8.3
**CA-CDI**					
Number of CA-CDI cases	1,025	1,069	1,059	1,425	1,409
Rate per 1,000 patient admissions	1.43	1.43	1.40	1.51	1.47
Rate per 10,000 patient days	1.86	1.82	1.68	1.87	1.81
Number of reporting hospitals	70	70	70	88	86
**All-cause mortality rate**					
Number of deaths	15	16	10	16	16
All-cause mortality rate per 100 cases (%)^b^	9.6	7.4	5.8	6.4	6.9

Abbreviations: *C. difficile*, *Clostridioides difficile*; CA, community-associated; CDI, *Clostridioides difficile* infections; HA, healthcare-associated

^a^ There was no resistance to tigecycline, vancomycin or metronidazole in *C. difficile* isolates submitted to the National Microbiology Laboratory 2019–2023

^b^ Mortality data are collected during the two-month period (March and April of each year) for adults (aged 18 years and older) and year-round for children (aged one year to younger than 18 years old). Among paediatric patients, there was no death attributable to healthcare-associated *C. difficile* infection

**Source of infection:** Stratified by the source of infection, HA-CDI rates decreased from 3.89 per 10,000 patient days in 2020 to 3.60 in 2021, followed by a period of relative stability through 2024 (3.69 per 10,000 patient days), Overall, no significant trend was observed (*p*=1.00) (Table 2). The CA-CDI rates remained stable over the five-year period. After holding constant at 1.43 per 1,000 patient admissions in 2020 and 2021, the rate was lowest in 2022 (1.40 per 10,000 patient days) before a small increase to 1.47 by 2024 (*p*=0.61) (Table 2).

Regionally, HA-CDI rates per 10,000 patient days fluctuated across all regions between 2020 and 2024, with no significant trend (Western range: 3.00–3.46, *p*=1.00; Central range: 3.48–3.82, *p*=0.09; Eastern range: 3.19–3.56, *p*=0.22). For CA-CDI, rates per 1,000 patient admissions remain highest in the Central region, with a downward trend from 2020 and 2024 (range: 1.54–1.70, *p*=0.11), while the Western region showed an upward trend, from 1.11 to 1.45 with a peak of 1.61 in 2023 (*p*=0.46). The CA-CDI rate in the Eastern region remained relatively stable, with a minor increase from 0.95 to 1.12 per 1,000 patient admissions (*p*=0.22). This indicates no statistically significant shift over the five-year period (Supplemental material).

**Hospital types:** The HA-CDI rates per 10,000 patient days were consistently higher in adult (range: 3.63–3.89) and paediatric hospitals (range: 3.34–3.97), with lower rates than observed in mixed hospitals (range: 2.56–3.09). The CA-CDI rates per 1,000 patient admissions were higher in adult (range: 1.74–1.82) and mixed hospital (range: 1.31–1.67), with lower rates observed in paediatric hospitals (range: 0.35–0.68) between 2020 and 2024 (Supplemental material). Stratified by hospital size, rates of HA-CDI were generally highest among large (range: 3.28–3.82), followed by medium (range: 3.20–3.60) and small size hospitals (range: 2.68–2.98). Rates of CA-CDI per 1,000 patient admissions were similar for large sized hospitals (range: 1.38–1.85) and medium sized hospitals (range: 1.39–1.52) and lower for small sized hospitals (range: 0.82–1.32), which follows a similar trend as HA-CDI (Supplemental material).

**30-day all-cause mortality:** Overall 30-day all-cause CDI mortality remained stable from 2020 to 2024 (range: 8.0–9.0, *p*=0.09) (Table 2). There was no significant difference in 30-day all-cause mortality between HA-CDI (8.3%) and CA-CDI (6.9%) in 2024 (*p*=0.51).

**Antimicrobial resistance:** From 2020 to 2024, 24.7% (n=608/2,458) of CDI isolates were resistant to one or more tested antimicrobials. The proportion of *C. difficile* isolates resistant to moxifloxacin fluctuated between 6.1% and 9.0%, with an average of 7.0% and 6.5% in 2024 (**Table 3**). Clindamycin resistance in HA and CA-CDI populations fluctuated from 2020 to 2024, with 2024 exhibiting the highest resistance rates at 34.0% and 30.7%, respectively (Supplemental material). None of 2,458 isolates tested was resistant to metronidazole, vancomycin or tigecycline.

**Table 3:** *Clostridioides difficile* antimicrobial resistance data, Canada, 2020–2024^a,b^

Antibiotic	Number of isolates and % resistance (per year)									
	2020		2021		2022		2023		2024	
	n	%	n	%	n	%	n	%	n	%
Clindamycin	62	17.0	67	12.4	101	22.7	69	13.1	192	33.0
Moxifloxacin	24	6.6	49	9.0	31	7.0	32	6.1	38	6.5
Rifampin	3	0.8	9	1.7	4	0.9	4	0.8	6	1.0
Total number of isolates tested^c^	365	N/A	542	N/A	444	N/A	525	N/A	582	N/A

Abbreviation: N/A, not applicable

^a^ *Clostridioides difficile* infection isolates are collected for resistance testing during the two-month period (March and April of each year) for adults (aged 18 years and older) and year-round for children (aged one year to younger than 18 years old) from admitted patients only

^b^ There was no resistance to tigecycline, vancomycin, or metronidazole in *C. difficile* isolates submitted to the National Microbiology Laboratory 2019–2023

^c^ Total reflects the number of isolates tested for each of the antibiotics listed above

**Molecular typing:** From 2020 to 2024, the five most prevalent ribotypes of isolates from HA-CDI cases were 106, 014, 020, 002 and 027, with overall prevalences of 15.9%, 8.7%, 7.0%, 5.7% and 4.8%, respectively, while the five most prevalent ribotypes of isolates from CA-CDI were 106, 014, 020, 002 and 015, with overall prevalences of 15.9%, 8.6%, 6.3%, 5.2% and 4.2%. From 2020 to 2024, the prevalence of RT027 associated with NAP1 decreased from 5.9% to 3.4% in HA-CDI but increased from 1.3% to 4.0% in CA-CDI (Supplemental material).

#### Methicillin-resistant *Staphylococcus aureus* bloodstream infections

Between 2020 and 2024, overall MRSA BSI rates remained stable, ranging from 0.99 to 1.16 infections per 10,000 patient days. The rate was lowest in 2022; however, no significant trend over time was observed (*p*=0.99) (**Table 4**). The median age among MRSA BSI patients was 57 years (IQR: 41–71), with women accounting for 36.6% of cases (Supplemental material).

**Table 4:** Methicillin-resistant *Staphylococcus aureus* bloodstream infections data, Canada, 2020–2024

MRSA BSI data	Year				
	2020	2021	2022	2023	2024
**All cases**					
Number of MRSA BSIs	868	872	835	914	993
Rate per 1,000 patient admissions	0.88	0.84	0.80	0.87	0.91
Rate per 10,000 patient days	1.16	1.11	0.99	1.11	1.16
Number of reporting hospitals	81	81	81	82	77
**All-cause mortality rate^a^**					
Number of deaths	145	164	164	175	187
All-cause mortality rate per 100 cases	16.7	18.8	20.2	19.1	19.0
**HA-MRSA BSI**					
Number of HA-MRSA BSIs	323	348	347	378	415
Rate per 1,000 patient admissions	0.33	0.34	0.33	0.36	0.38
Rate per 10,000 patient days	0.43	0.44	0.41	0.46	0.49
Number of reporting hospitals	81	81	81	82	77
**All-cause mortality rate^a^**					
Number of deaths	62	86	81	94	94
All-cause mortality rate per 100 cases	19.2	24.7	23.7	24.9	22.8
**CA-MRSA BSI**					
Number of CA-MRSA BSIs	480	471	453	528	560
Rate per 1,000 patient admissions	0.49	0.46	0.44	0.51	0.52
Rate per 10,000 patient days	0.65	0.61	0.55	0.66	0.67
Number of reporting hospitals	80	80	80	81	76
**All-cause mortality rate^a^**					
Number of deaths	72	71	79	80	91
All-cause mortality rate per 100 cases	15.0	15.1	17.8	15.2	16.4

Abbreviations: CA, community-associated; HA, healthcare-associated; MRSA BSI, methicillin-resistant *Staphylococcus aureus* bloodstream infection

^a^ Based on the number of cases with associated 30-day outcome data

**Source of infection:** Rates for CA-MRSA BSI did not change significantly (*p*=0.46) between 2020 (0.65 infections per 10,000 patient days) and 2024 (0.67 per 10,000 patient days). Healthcare-associated-MRSA BSI rates remained stable (range: 0.42–0.47 infections per 10,000 patient days) (Table 4).

Rates for HA-MRSA BSIs have remained stable across all regions (Western range: 0.46–0.58; Central range: 0.36–0.45; Eastern range: 0.36–0.58; Northern range: zero infections per 10,000 patient days) (Supplemental material). The CA-MRSA BSI rates remained stable across all regions except for in the East where there was a significant increase, from 0.34 in 2020 to 0.67 infections per 10,000 patient days in 2024 (*p*=0.03) (Western range: 0.70–0.83; Central range: 0.43–0.63; Eastern range: 0.34–0.67; Northern range: zero infections per 10,000 patient days) (Supplemental material). In 2024, CA-MRSA and HA-MRSA BSI rates were highest in Western Canada (0.71 and 0.57 infections per 10,000 patient days, respectively) (Supplemental material).

**Hospital types:** Both HA- and CA-MRSA BSI rates remained higher over time in adult and mixed hospitals from 2020 to 2024 (HA-MRSA: adult range: 0.50–0.65; mixed range: 0.36–0.47; CA-MRSA: adult range: 0.70–0.86; mixed range: 0.55–0.79, with lower rates observed in adult hospitals with a NICU (HA-MRSA range: 0.30–0.46; CA-MRSA range: 0.24–0.47 infections per 10,000 patient days), paediatric (HA-MRSA range: 0.30–0.43; CA-MRSA range: 0.32–0.43 infections per 10,000 patient days) and paediatric-OB hospitals (HA-MRSA and CA-MRSA range: 0.04–0.22 infections per 10,000 patient days) (Supplemental material). Stratified by hospital size, both HA-and CA-MRSA BSI rates were generally highest among medium (201–499 beds; HA-MRSA range: 0.38–0.47; CA-MRSA range: 0.64–0.81) and large size hospitals (500 or more beds; HA-MRSA range: 0.41–0.60; CA-MRSA range: 0.52–0.73) (Supplemental material). There were no significant trends over time observed by hospital type or size during this reporting period (*p*>0.05).

**30-day all-cause mortality:** Thirty-day all-cause mortality remained stable from 2020 to 2024 (range: 16.7–20.2) (Table 4). In 2024, 30-day all-cause mortality was significantly higher for HA-MRSA (22.8%) compared to CA-MRSA (16.4%) (*p*=0.02).

**Antimicrobial resistance:** Clindamycin resistance among MRSA isolates decreased significantly from 33.4% to 27.6% between 2020 and 2024 (*p*<0.01) (**Table 5**). Since 2020, the proportion of MRSA isolates resistant to erythromycin has stayed relatively stable and high at around 68% in relation to other antibiotics tested. Resistance to tetracycline significantly increased from 6.6% in 2020 to 10.1% in 2024 (*p*=0.02). All tested MRSA BSI isolates from 2020 to 2024 were susceptible to linezolid, daptomycin and vancomycin.

**Table 5:** Methicillin-resistant *Staphylococcus aureus* bloodstream antimicrobial resistance data, Canada, 2020–2024^a^

Antibiotic	Year									
	2020		2021		2022		2023		2024	
	n	%	n	%	n	%	n	%	n	%
Ciprofloxacin	460	65.6	490	65.8	415	66.5	512	63.4	566	61.6
Clindamycin	234	33.4	220	29.5	157	25.2	186	23.0	254	27.6
Daptomycin	0	0	0	0	0	0	0	0	0	0
Erythromycin	507	72.3	510	68.5	428	68.6	543	67.2	629	68.4
Gentamicin	22	3.1	35	4.7	20	3.2	33	4.1	44	4.8
Linezolid	0	0	0	0	0	0	0	0	0	0
Rifampin	6	0.9	10	1.3	5	0.8	9	1.1	12	1.3
Trimethoprim/sulfamethoxazole	16	2.3	32	4.3	36	5.8	20	2.5	20	2.2
Tetracycline	46	6.6	63	8.5	52	8.3	71	8.8	93	10.1
Tigecycline	1	0.1	6	0.8	5	0.8	5	0.6	13	1.4
Vancomycin	0	0	0	0	0	0	0	0	0	0
Total number of isolates tested^b,c^	701	N/A	745	N/A	624	N/A	808	N/A	919	N/A

Abbreviation: N/A, not applicable

^a^ All methicillin-resistant *Staphylococcus aureus* bloodstream infection (MRSA) isolates from 2020 to 2024 submitted to the National Microbiology Laboratory were susceptible to nitrofurantoin

^b^ In some years, the number of isolates tested for resistance varied by antibiotic

^c^ Total reflects the number of isolates tested for each of the antibiotics listed above

Comparing isolates from HA-MRSA with CA-MRSA cases, clindamycin resistance was consistently higher among isolates from HA-MRSA each year from 2020 (35.0%, n=89/254 vs. 31.1%, n=117/376) to 2024 (32.2%, n=125/388 vs. 24.3%, n=117/481) (Supplemental material). There were no other notable differences in antibiotic resistance patterns by MRSA BSI case type.

**Molecular typing:** Between 2020 and 2024, the proportion of spa types identified as t002, most commonly associated with HA-MRSA, continued to decrease from 15.7% of all isolates in HA-MRSA cases in 2020 to 8.2% in 2024 (*p*<0.01) (Supplemental material). Spa type t008, most commonly associated with CA-MRSA, accounted for the largest proportion of isolates identified in both CA-MRSA (33.7%) and HA-MRSA (47.4%) cases (Supplemental material). Among CA-MRSA, the proportion of t008 increased from 45.4% in 2020 to 47.2% in 2024 (*p*=0.59). In contrast, spa type t008 among HA-MRSA significantly increased from 28.0% in 2020 to 35.4% in 2024 (*p*<0.01).

### Vancomycin-resistant *Enterococcus* bloodstream infections

From 2020 to 2024, VRE BSI rates significantly increased from 0.30 to 0.42 infections per 10,000 patient days (*p*=0.01) (**Table 6**). The median age among patients with VRE BSI was 63 years (IQR: 51–71) and women accounted for 38.8% of VRE BSI cases (Supplemental material).

**Table 6:** Vancomycin-resistant *Enterococcus* bloodstream infections data, Canada, 2020–2024^a^

VRE BSI data	Year				
	2020	2021	2022	2023	2024
Number of VRE BSIs	224	251	305	318	370
Rate per 1,000 patient admissions	0.23	0.24	0.29	0.29	0.33
Rate per 10,000 patient days	0.30	0.32	0.36	0.37	0.42
Number of reporting hospitals	81	80	80	85	85
**All-cause mortality rate^b^**					
Number of deaths	82	84	117	118	142
All-cause mortality rate per 100 cases	36.6	33.5	38.5	37.1	38.4

Abbreviation: VRE BSI, vancomycin-resistant *Enterococcus* bloodstream infection

^a^ Due to the low number of community-associated VRE BSI cases reported each year, this table presents data for all cases combined (healthcare-associated and community-associated)

^b^ Based on the number of cases with associated 30-day outcome data

Note: Aggregate mortality data reported in-text due to fluctuations in the small numbers of VRE BSI deaths reported each year

**Source of infection:** Vancomycin-resistant *Enterococcus* BSIs were predominantly HA, as 90.3% (n=1,325/1,468) of VRE BSIs reported from 2020 to 2024 were acquired in a healthcare facility. Stratified by source of infection, HA-VRE BSI rates significantly increased from 2020 to 2024 from 0.28 to 0.39 infections per 10,000 patient days (*p*=0.03) (Supplemental material). Community-acquired-VRE BSI rates remained low and stable over time (range: 0.02–0.04 infections per 10,000 patient days).

Regionally, VRE BSI rates in Western and Central Canada significantly increased between 2020 and 2024 from 0.39 to 0.54 infections per 10,000 patient days (*p*=0.04) and 0.29 to 0.38 infections per 10,000 patient days (*p*=0.04), respectively. No significant increasing trend was observed in Eastern Canada (range: 0.00–0.12 infections per 10,000 patient days, *p*=0.11) (Supplemental material).

**Hospital types:** Stratified by hospital type, VRE BSI rates remained highest in adult hospitals from 2020 to 2024 (range: 0.43–0.57 infections per 10,000 patient days). From 2020 to 2024, VRE BSI rates in paediatric hospitals were low (range: 0.00–0.11 infections per 10,000 patient days) and there were no VRE BSIs in paediatric-OB hospitals. In 2024, VRE BSI rates were highest in large hospitals (500 or more beds) at 0.56 infections per 10,000 patient days, followed by medium hospitals (201–499 beds) at 0.31 infections per 10,000 patient days and small hospitals (1–200 beds) at 0.20 infections per 10,000 patient days. A significant increasing trend in VRE BSI rates was observed over time in large hospitals (500 or more beds, *p*=0.01), but not in medium hospitals (201–499 beds, *p*=0.50) and small hospitals (1–200 beds, *p*=0.07). The incidence rates for HA-VRE BSI by region, hospital type and hospital size are presented in Supplemental material.

**30-day all-cause mortality:** All-cause mortality remained high and stable over time from 2020 to 2024 (range: 33.5–38.5) (*p*=0.23) (Table 6).

**Antimicrobial resistance:** Resistance to last resort antimicrobials such as daptomycin and linezolid has remained low from 2020 to 2024. Daptomycin resistance rates were relatively stable at 4.5% (n=6/134) in 2020 to 4.0% (n=13/328) in 2024, while linezolid resistance rates were 0.7% (n=1/134) in 2020 and 1.2% (n=4/328) in 2024 (**Table 7**).

**Table 7:** Antimicrobial resistance of *Enterococcus faecium* isolates, Canada, 2020–2024^a^

Antimicrobial	Year									
	2020		2021		2022		2023		2024	
	n	%	n	%	n	%	n	%	n	%
Ampicillin	132	98.5	166	98.8	199	97.5	223	97.8	320	97.6
Chloramphenicol	28	20.9	51	30.4	34	16.7	38	16.7	59	18.0
Ciprofloxacin	132	98.5	166	98.8	203	99.5	226	99.1	322	98.2
Daptomycin	6	4.5	5	3.0	4	2.0	4	1.8	13	4.0
Erythromycin	128	95.5	159	94.6	199	97.5	221	96.9	304	92.7
High-level gentamicin	36	26.9	34	20.2	39	19.1	42	18.4	85	25.9
Levofloxacin	131	97.8	166	98.8	202	99.0	226	99.1	323	98.5
Linezolid	1	0.7	3	1.8	6	2.9	1	0.4	4	1.2
Nitrofurantoin	56	41.8	131	78.0	143	70.1	141	61.8	187	57.0
Penicillin	133	99.3	166	98.8	200	98.0	223	97.8	320	97.6
Quinupristin/dalfopristin	9	6.7	8	4.8	16	7.8	34	14.9	41	12.5
Rifampicin	115	85.8	155	92.3	188	92.2	211	92.5	311	94.8
High-level streptomycin	29	21.6	48	28.6	51	25.0	63	27.6	106	32.3
Tetracycline	89	66.4	134	79.8	180	88.2	186	81.6	258	78.7
Tigecycline	0	0	0	0	0	0	0	0	2	0.6
Vancomycin	130	97.0	163	97.0	203	99.5	228	100	322	98.2
Total number of isolates^b^	134	-	168	-	204	-	228	-	328	-

^a^ Due to the low number of community-associated vancomycin-resistant *Enterococcus* bloodstream infection cases reported each year, this table presents data for all cases combined (healthcare-associated and community-associated)

^b^ Total reflects the number of isolates tested for each of the antibiotics listed above

Note: Antimicrobials presented are for surveillance purposes. Please refer to Clinical & Laboratory Standards Institute (CLSI) for appropriate treatment of bloodstream infection *Enterococcus* infections (CLSI M100 ED34:2024)

**Molecular typing:** From 2020 to 2024, most VRE BSI isolates were identified as *E. faecium*. *Enterococcus faecalis* was detected infrequently, with one isolate identified in 2020 (0.7%), 2021 (0.6%) and 2022 (0.5%); three isolates in 2023 (1.3%); and seven isolates in 2024 (2.1%) (Supplemental material).

Although VanA remained predominant, an increasing proportion of *E. faecium* isolates harboured VanB, rising from 3.0% (n=4) in 2020 to 6.7% (n=22) in 2024 (*p*=0.60) (Supplemental material).

Four predominant sequence types were identified among *E. faecium* isolates from 2020 to 2024, with a notable shift in their distribution observed over time (Supplemental material). A significant decrease in ST1478 was observed, declining from 19.5% (n=26/133) in 2020 to 2.2% (n=7/320) in 2024 (*p*<0.01). The proportion of ST17 isolates also decreased; from 33.8% (n=45/133) in 2020 to 25.0%, (n=80/320) in 2024 (*p*=0.16). In contrast, ST117 increased from 10.5% (n=14/133) in 2020 to 22.2% (n=71/320) in 2024 (*p*=0.02), while ST80 increased from 16.5% (n=22/133) to 30.6% (n=98/320) over the same period (*p*=0.02). A statistically significant increasing trend in ST80 was observed from 2020 to 2024, and by 2024, ST80 had become the predominant sequence type among all tested isolates (*p*=0.04).

#### Carbapenemase-producing *Enterobacterales* and *Acinetobacter baumannii*

From 2020 to 2024, CPE infection rates have remained low compared to other HAIs in Canada, although there has been a significant increase in the rates over this period (0.05–0.20 infections per 10,000 patient days, *p*=0.03) (**Table 8**). The number of CPA infections were very low with eight or fewer cases per year between 2020 and 2024 (total n=22). The median age for CPE infections was 65 years and 42.6% of cases were female (Supplemental material).

**Table 8:** Carbapenemase-producing *Enterobacterales* data, Canada, 2020–2024

CPE data	Year				
	2020	2021	2022	2023	2024
Number of CPE infections	39	67	101	169	218
Infection rate per 1,000 patient admissions	0.04	0.07	0.09	0.13	0.16
Infection rate per 10,000 patient days	0.05	0.09	0.12	0.17	0.20
Number of reporting hospitals	81	81	85	102	105
**All-cause mortality rate**					
Number of CPE infection deaths	7	13	17	25	33
All-cause mortality rate per 100 cases	18	19.4	16.8	14.8	15.1

Abbreviation: CPE, Carbapenemase-producing *Enterobacterales*

Note: All-cause mortality only includes CPE infections that have a 30-day outcome available

From 2020 to 2024, the majority (51.7%; n=307/594) of CPE infections of the were identified in Western Canada, followed by 44.3% (n=263/594) in Central Canada and 4.0% (n=307/594) in Eastern Canada (Supplemental material). From 2020 to 2024, large hospitals (500 or more beds) generally reported the highest rates of CPE infections (0.06–0.28 infections per 10,000 patient days) compared to small hospitals (fewer than 200 beds) (0.03–0.09 infections per 10,000 patient days). During this period, 26.7% (n=119/445) of CPE-infected patients reported travel outside of Canada and of those, 79.1% (n=83/105) received medical care while abroad. The majority of CPE infections were acquired domestically, with 86.8% (n=446/514) of CPE infections acquired in Canada and 80% (n=357/446) acquired within a Canadian acute care hospital between 2020 and 2024. The number of CPE infections acquired in the community has also increased from 12.9% (n=4/31) in 2020 to 20.6% (n=37/180) in 2024.

**Organisms:** Of all isolates submitted (infections and colonizations), the top four carbapenemase producing organisms during 2024 were *Escherichia coli* (42.1%), *Klebsiella pneumoniae* (16.3%), *Enterobacter cloacae* (15.8%) and *Citrobacter freundii* (14.6%). From 2020 to 2024, there has been an increase in the proportion of *E. coli*-producing carbapenemases (39.3%–42.1%) and a decrease in the proportion of *K. pneumoniae* (19.9%–16.34%) and *E. cloacae* (18.1%–15.8%) producing carbapenemases (Supplemental material). The predominant carbapenemases, in order identified in Canada have not changed over the study period and were *K. pneumoniae* carbapenemase (KPC), New Delhi metallo-β-lactamase (NDM) and oxacillinase-48 (OXA-48), accounting for over 90% of identified carbapenemases from 2020 to 2024 (**Table 9**). Historically, KPC has been the most commonly identified carbapenemase in Canada; however, the proportion of KPC and NDM have been continually trending closer and were almost equal in 2024. Over time a significant decrease in KPC and an increase in NDM, OXA-48, and NDM+OXA-48 was observed (*p*≤0.002).

**Table 9:** Carbapenemases identified in carbapenemase-producing *Enterobacterales* isolates, Canada, 2020–2024

Carbapenemases identified^a^	Year									
	2020		2021		2022		2023		2024	
	n	%	n	%	n	%	n	%	n	%
KPC	98	40	178	50.1	214	45.3	397	38.4	525	38.4
NDM	80	32.7	85	23.9	131	27.8	350	33.8	458	33.5
OXA-48	48	19.6	57	16.1	94	19.9	194	18.7	277	20.3
SME^b^	2	0.8	1	0.3	0	0	2	0.2	3	0.2
NDM/OXA-48	9	3.7	12	3.4	14	3	57	5.5	69	5.0
GES	0	0	1	0.3	0	0	0	0	0	0
IMP	1	0.4	2	0.6	2	0.4	1	0.1	7	0.5
IMI/NMC	7	2.9	15	4.2	3	0.6	13	1.3	7	0.5
VIM	0	0	1	0.3	6	1.3	4	0.4	3	0.2
Other	0	0	3	0.8	8	1.7	17	1.6	18	1.3
Total number of isolates tested^c^	245	N/A	355	N/A	427	N/A	1,035	N/A	1,367	N/A

Abbreviations: CPE, carbapenemase-producing *Enterobacterales*; GES, Guiana extended-spectrum β-lactamase; IMI, imipenem-hydrolyzing β-lactamase; IMP, active-on-imipenem; KPC, *Klebsiella pneumoniae*; carbapenemase; NDM, New Delhi metallo-β-lactamase; NMC, not metalloenzyme carbapenemase; N/A, not applicable; OXA-48, oxacillinase-48; SME, *Serratia marcescens* enzymes; VIM, Verona integron-encoded metallo-β-lactamase

^a^ Includes data for all CPE isolates submitted (infections and colonisations)

^b^ Only found in *Serratia marcescens*

^c^ Some isolates contain multiple carbapenemases, therefore the total number of isolates tested and the number of carbapenemases indicated may not match. *Acinetobacter baumanii* were not included in this table

**30-day all-cause mortality:** All-cause mortality for CPE infections fluctuated between 2020 and 2024, with a mean of 16.8% (Table 8).

**Antibiotic resistance:** In all years, NDM producing isolates were predominantly extensively drug-resistant (XDR) (range: 83.8%–91.8%). Conversely, in 2020, 37.5% of OXA-48-like producers were XDR compared to 2024 where 16.7% are XDR, showing an overall downward trend in resistance. *Klebsiella pneumoniae* carbapenemase has been more equally distributed throughout 2020–2024 for either XDR (range: 40.8–56.3) or multidrug-resistant (MDR) (range: 31.1–52.2). When examining resistance among the top three carbapenemases, we noted that there was an increase in resistance to all aminoglycosides from 2021 to 2024 in KPC producers (Supplemental material). New Delhi metallo-β-lactamase producers showed increasing trends in Tobramycin. Conversely, among OXA-48-like producers, there was a decline in resistance to aztreonam, doxycycline, minocycline, trimethoprim/sulfamethoxazole, carbapenems, tobramycin and gentamicin. This agrees with observations that fewer OXA-48-like producers were XDR or MDR over time. From 2020 to 2024, the overall resistance in KPC, NDM and OXA-48-like producers to ertapenem was 75.1%, 97.8% and 63.3%, respectively, and for meropenem was 54.5%, 92.1% and 14.5%, respectively. Resistance to newer combination drugs, such as meropenem/vaborbactam, imipenem/relebactam and ceftazidime/avibactam among KPC producers (0.5%, 1.4% and 1.3%) and OXA-48-like producers (10.2%, 11.5% and 0.3%), was low. Meropenem/vaborbactam and imipenem/relebactam resistance in NDM producers ranged from 61.3% to 69.5% and 86.3% to 93.0%, respectively, over five years.

#### Candidozyma auris (Candida auris)

Ninety-six percent (n=105/109) of CNISP hospitals participate in *C. auris* surveillance. Between CNISP and the National Microbiology Laboratory surveillance, a total of 43 isolates (colonizations and infections) has been reported from 2020 to 2024: 19 (44%) from CNISP hospitals and 24 (56%) from other hospital laboratories. The number of *C. auris* cases detected per year was four in 2020, three in 2021, 12 in 2022, 10 in 2023 and 14 in 2024. Twelve (27.9%) of the total cases were from Western Canada, 29 (67.4%) cases were from Central Canada and two (4.7%) cases were from Eastern Canada. Of the 43 *C. auris* isolates, 19.5% were resistant to amphotericin B and 90.2% were resistant to fluconazole (**Table 10**). The first identification of echinocandin-resistant *C. auris* in Canada occurred in 2024; this isolate was to fluconazole and micafungin. Between 2020 to 2024, 22% of isolates were MDR (resistant to two classes of antifungals). Based on available travel information (n=24), 33.3% reported no travel while 66.7% either received health care or travelled abroad (Table 8). Of the 15 *C. auris* patients who received health care abroad, 10 (66.7%) had known carbapenemase-producing organism status and four (40%) were carbapenemase-producing organism positive.

**Table 10:** Antifungal resistance of *Candidozyma auris* isolates, Canada, 2020–2024

Isolate or patient characteristics^a^	Number of cases		
	n		%
**Antifungal resistance of *Candidozyma auris* isolates (n=41)**			
Fluconazole	37	90.2	
Amphotericin B	8	19.5	
Multidrug resistant	9	22.0	
Micafungin	1	2.4	
**Travel history (n=24)**			
Receipt of health care abroad	15	62.5	
Travel abroad (receipt of health care unknown)	1	4.2	
No travel reported	8	33.3	

^a^ 2/43 isolates did not have antifungal susceptibility results and 19/43 cases had unknown travel history

## Discussion

Between 2020 and 2024, CNISP surveillance data indicate HAI infection rates in Canada have remained relatively stable for CDI (−3.5% change) and MRSA BSI (no change); however, rates have increased for VRE BSI and CPE infections (40% and 300%, respectively). A total of 43 *C. auris* isolates were identified from 2020 to 2024 with the number of cases increasing each year.

*Clostridioides difficile* infections between 2020 to 2024, overall CDI rates in the CNISP network were stable, with HA-CDI rates ranging from 3.60 to 3.89 per 10,000 patient days and CA-CDI rates ranging from 1.40 to 1.51 per 1,000 patient admissions. When compared to pooled WHO regional rates from 2016 to 2024, overall CNISP CDI rates were lower than the pooled North America rate (6.23 per 10,000 patient days), higher than Latin America rate (3.09 per 10,000 patient days) and similar to Western Pacific (3.90 per 10,000 patient days) and European (3.57 per 10,000 patient days) rates ((21)). At the country level, in contrast to the stable CDI rates observed in the CNISP network, the United Kingdom has recently reported an approximate 33% increase in rate in 2023/2024 compared to 2020/2021, following a previously stable trend ((22)).

While CDI results remained stable, the 30-day all-cause mortality rates among CDI patients decreased during the reporting period. These declining mortality rates occurred in both CA-CDI and HA-CDI and are likely, in part, associated with the decreased prevalence of the hypervirulent NAP1 ((23)). Improved diagnosis and management may also have reduced case fatality rates.

*Clostridioides difficile* infection AMR is less common in Canada than in the United States or globally ((24)). In a representative sample of Canadian acute care hospitals, from 2020 to 2024, we saw a stabilization in moxifloxacin resistance in both HA- and CA-CDI populations with an average resistance of 7.0%. The decrease in moxifloxacin resistance from 24.8% in 2015 is concordant with an overall decrease in the prevalence of RT027 (NAP1). Furthermore, moxifloxacin resistance remained lower (6.5% in 2024) than previously published weighted pooled resistance data for North America (44.0%) and Asia (33.0%) ((25,26)). The decline in the prevalence of RT027 has been replaced with a concomitant increase in the prevalence of RT106, RT014 and RT020, consistent with trends observed in the United States ((23,27)). Additionally, the emergence of RT106 now found worldwide, presents additional challenges as this strain has been shown to produce more spores, have higher rates of recurrence, and be highly resistant to erythromycin, clindamycin, fluoroquinolones and third-generation cephalosporins. The potential emergence of resistant ribotypes warrants further surveillance, monitoring and investigation ((27,28)).

Methicillin-resistant *Staphylococcus aureus* remains a high priority pathogen due to its estimated burden of disease and mortality rate ((29,30)). Between 2020 and 2024, MRSA BSI rates in the CNISP network remained stable (0.99–1.16 infections per 10,000 patient days). Similarly, surveillance data measuring population based estimated incidence from the European Union/European Economic area showed no significant trends among MRSA BSIs during this period ((31)).

From 2020 to 2024, HA-MRSA BSI rates in CNISP (0.41–0.49 infections per 10,000 patient days), were considerably higher than rates reported in Australian public hospitals between 2020 and 2024 (0.09–0.13 infections per 10,000 patient days); however, broader CNISP case definitions likely capture more cases with indirect healthcare exposures not included in the Australian case definition ((32,33)).

During this reporting period, CA-MRSA BSI rates have slightly increased; however, this trend was not significant. Increases in CA-MRSA BSI have been reported in other jurisdictions, suggesting an expanding community reservoir of MRSA in Canada and globally ((34)).

The CNISP 30-day all-cause mortality rates for MRSA BSI (HA: 19.2%–24.90%; CA: 15.0%–17.8%) were lower than those previously reported in the United States (HA: 29%; CA: 18%) ((35)). Differences may stem from CNISP’s strict 30-day mortality cut-off versus undefined United States time frames or from variances in healthcare systems, infection prevention strategies and population characteristics ((35,36)).

A significant decrease in clindamycin resistance among MRSA BSI isolates between 2020 and 2024 coincided with shifts in MRSA spa types. The proportion of spa type t002 (commonly HA-MRSA) declined, while spa type t008 (historically CA-MRSA) increased. Notably, t008 rose among CA-MRSA isolates (45.4%–47.2%) and HA-MRSA isolates (28%–35.4%). The growing prevalence of traditionally CA clones in hospitals emphasizes the need for ongoing surveillance and tailored infection prevention strategies, as well as continued monitoring of antimicrobial resistance to guide treatment and mitigate MRSA burden in both healthcare and community settings. Populations at increased risk for CA-MRSA infection include children, athletes, incarcerated individuals, older adults with comorbidities, people who inject drugs and people experiencing homelessness that use public facilities including shelters ((37–40)). Injection drug use may represent an emerging risk factor for CA-MRSA ((38–40)). Targeted strategies such as MRSA screening and decolonization in high-risk populations may contribute to reducing the burden of MRSA BSIs ((37–40)).

Vancomycin resistance related to VRE BSI has been shown to be associated with higher mortality rates and longer hospital stays, making it a significant public health concern ((41–43)). The rate of VRE BSIs has increased year-over-year among CNISP-participating hospitals and reached an all-time high in 2024 (0.42 infections per 10,000 patient days). The highest VRE BSI rates were observed among Western and Central Canadian adult hospitals with 500 or more beds. The success of certain sequence types likely contributed to the increasing burden of VRE BSIs in CNISP-participating hospitals. In 2024, the prevalence of the previously dominant clone ST17 decreased to 25.0%, while ST80 emerged as the predominant clone, accounting for 30.6% of isolates. Compared to other sequence types, a distinct association has been identified between ST80 and the VanB gene. This association of VanB genes harboured predominantly among ST80 isolates has also been documented in recent studies related to VanB outbreaks in Sweden and Denmark ((44,45)). Increasing trends have been noted in other jurisdictions, such as Germany and India, which may be associated, in part, with the introduction and spread of new clones, differences in antibiotic prescription practices, and gaps in infection prevention practices ((46–50)). Because all-cause 30-day mortality remains high and most VRE BSI cases reported by CNISP-participating hospitals were HA, continued surveillance and targeted infection prevention measures in hospital are of utmost importance. Furthermore, treatment options for VRE BSIs are limited and require the use of daptomycin and linezolid, which are classified as last-resort reserve antibiotics under WHO’s AWaRe classification ((51)). Antibiotic susceptibility data up to 2024 show that the great majority of VRE BSI isolates remain susceptible to daptomycin and linezolid (more than 95%); however, continued monitoring is needed to capture any changes in AMR trends over time.

Carbapenemase-producing *Enterobacterales* infections are a significant threat to public health as they are becoming increasingly prevalent in healthcare environments worldwide and are associated with high mortality and limited treatment options ((52–55)). The Centers for Disease Control and Prevention and WHO have classified CPE as one of the most urgent AMR threats ((56,57)). Among CNISP-participating hospitals, the number of CPE infections increased more than five-fold from 2020 to 2024 and the increased infection rate was significant (*p*=0.03). Data on the incidence of CPE infections in other countries, such as Denmark, Italy, Switzerland and the United Kingdom, have also shown an increasing incidence of CPE infections ((30,58–61)). From 2020 to 2024, 86.8% of CPE infections were domestically acquired and 80% were acquired in a Canadian acute care hospital, emphasizing the importance of continued surveillance and rigorous, multi-layered infection control measure strategies, including screening patients with a previous hospitalization (domestic or abroad). Data from new antimicrobial drugs such as ceftazidime/avibactam show low resistance to carbapenemases such as KPC and OXA-48. In agreement with several other studies drugs such as imipenem/relebactam, meropenem/vaborbactam and ceftazidime/avibactam are not affective on NDM producers where high resistance is often observed. As increasing trends in NDM prevalence is observed testing of newer agents affective to this carbapenemase are needed.

### Candidozyma auris

*Candidozyma auris* is an emerging MDR fungus that can cause invasive infections and outbreaks, in which invasive infections have a very high mortality rate (15%–60%) ((62–64)). *Candidozyma auris* has also been detected in dozens of countries ((62–68)). Although still relatively rare in Canada, the number of cases increased from four cases in 2020 to 14 cases in 2024. The United States reported over 7,000 clinical cases in 2025 ((69)). Identifying *C. auris* in routine microbiology laboratories requires identification of *Candida* to the species level, which, even in CNISP hospitals, was performed for all isolates of *Candida* in 45% of laboratories in 2018 and 81% in 2024 ((70)). Treatment options are limited, as over 20% of identified *C. auris* isolates in Canada were MDR and additional resistance can develop during antifungal therapy ((71,72)). Rapid identification, screening for colonization in at-risk patients’ adherence to routine practices and additional precautions, and investigation of potential transmission are all required to reduce the transmission of *C. auris* in Canadian healthcare settings. With increasing detection of *C. auris* in Canada, continued reporting is critical to monitor the risk and identify epidemiological and microbiological trends.

### Strengths and limitations

The strengths of CNISP lie in its network size, collaborative nature, detailed data collection (epidemiological and laboratory), standardized procedures and frequent and routine data quality evaluation. Epidemiological data collected through CNISP include information available in patient medical charts related to clinical care as well as data collected by infection prevention and control programs. Although staff turnover in hospitals might have influenced the consistent application of CNISP case definitions during chart reviews, data collection was carried out by trained and experienced infection prevention and control professionals who receive regular refresher training on CNISP methodology and definitions. In addition, routine data quality assessments were conducted to support data accuracy and consistency. These data may also be affected by selection bias due to the exclusion of sites with missing or incomplete information during the study period. A further limitation of *C. auris* surveillance is that detailed epidemiologic information is only available for patients identified at CNISP participating hospitals.

Efforts to improve the quality and representativeness of Canadian HAI surveillance data are ongoing. Additionally, the enhanced hospital screening practices survey is conducted annually to contextualize changes in HAI rates in the CNISP network. Canadian Nosocomial Infection Surveillance Program also conducts point prevalence surveys to assess the burden and incidence of HAIs and antimicrobial use; the fourth point prevalence survey was conducted from February to March 2024 ((2)). To further improve representativeness and generalizability of national HAI benchmark rates, CNISP has launched a simplified dataset accessible to all acute care hospitals across Canada to collect and visualize annual HAI rate data and has 109 hospitals participating in the project. With the launch of the simplified dataset, CNISP’s coverage of acute care beds in Canada increased from 35% in 2020 to 49% in 2024, thereby improving representativeness across northern, community, rural and Indigenous populations ((73)). These and other detailed CNISP data, data exploration tools and analytics are available on the CNISP Health Infobase website ((73)).

### Conclusion

Surveillance findings from a national sentinel network of Canadian acute care hospitals indicate that rates of MRSA BSI and CDI have remained stable from 2020 to 2024, while rates of VRE BSI and CPE infections have increased. Few cases of *C. auris* were detected in Canada, but the numbers have increased. Continued monitoring of HAIs in Canada is vital to understanding trends in the data, to provide benchmark rates for national and international comparisons and to evaluate and create interventions and policy to improve the quality of healthcare in Canada. These data also continue to form one of the key evidence bases for monitoring rates of AMR in Canada, as we work towards meeting commitments outlined in the *Pan-Canadian Action Plan on Antimicrobial Resistance* ((16)).

## Appendix

Supplemental material and tables are available upon request to the author: cnisp-pcsin@phac-aspc.gc.ca

Surveillance case definitions and eligibility criteria, 2024

Table S1.0: Summary of patient characteristics for *Clostridioides difficile* infections (CDIs), carbapenemase-producing *Enterobacterales* (CPE) infections, methicillin-resistant *Staphylococcus aureus* (MRSA) bloodstream infections (BSIs), and vancomycin-resistant *Enterococcus* (VRE) BSIs, 2020–2024

Table S1.1: Cases and incidence rates of healthcare-associated and community-associated *Clostridioides difficile* infection by region, hospital type and hospital size, Canada, 2020–2024

Table S1.2: Antimicrobial resistance of healthcare-associated and community-associated *Clostridioides difficile* infection isolates, Canada, 2020–2024

Table S1.3: Number and proportion of common ribotypes of healthcare-associated and community-associated *Clostridioides difficile* infection cases, Canada, 2020–2024

Table S2.1: Cases and incidence rates of healthcare-associated and community-associated methicillin-resistant *Staphylococcus aureus* bloodstream infections by region, hospital type and hospital size, 2020–2024

Table S2.2: Antimicrobial resistance of healthcare-associated and community-associated methicillin-resistant *Staphylococcus aureus* bloodstream infection isolates, Canada, 2020–2024

Table S2.3: Number and proportion of select methicillin-resistant *Staphylococcus aureus* spa types (with corresponding epidemic types) identified

Table S3.1: Number of vancomycin-resistant *Enterococcus* bloodstream infections incidence rates by region, hospital type and hospital size, 2020–2024

Table S3.2: Number of healthcare-associated vancomycin-resistant *Enterococcus* bloodstream infections and incidence rates by region, hospital type and hospital size, 2020–2024

Table S3.3: Number and proportion of vancomycin-resistant *Enterococcus* bloodstream infections isolate types identified, 2020–2024

Table S3.4: Distribution of vancomycin-resistant *Enterococcus faecium* bloodstream sequence types, 2020–2024

Table S4.1: Number of carbapenemase-producing *Enterobacterales* infections and incidence rates by region, hospital type and hospital size, 2020–2024

Table S4.2: Number and proportion of main carbapenemase-producing pathogens identified

Table S4.3: Antimicrobial Susceptibility Testing for *Klebsiella pneumoniae* carbapenemase, 2020–2024

Table S4.4 Antimicrobial Susceptibility Testing for New Delhi metallo-β-lactamase, 2020–2024

Table S4.5: Antimicrobial Susceptibility Testing for OXA-48, Oxacillinase-48, 2020–2024
